# Peritoneal Dialysis Aggravates and Accelerates Atherosclerosis in Uremic *ApoE* ^−/−^ Mice

**DOI:** 10.1161/JAHA.123.034066

**Published:** 2024-07-09

**Authors:** Jamie Kane, Winnie G. Vos, Laura A. Bosmans, Bram W. van Os, Myrthe den Toom, Sanne Hoeksema‐Hackmann, Denise Moen‐de Wit, Marion J. Gijbels, Linda Beckers, Aldo Grefhorst, Johannes H. M. Levels, Lily Jakulj, Marc G. Vervloet, Esther Lutgens, Etto C. Eringa

**Affiliations:** ^1^ Department of Nephrology, Amsterdam Cardiovascular Sciences Amsterdam University Medical Centre Amsterdam the Netherlands; ^2^ Department of Physiology, Amsterdam Cardiovascular Sciences Amsterdam University Medical Centre Amsterdam the Netherlands; ^3^ Department of Medical Biochemistry, Amsterdam Cardiovascular Sciences, Amsterdam Immunity and Infection Amsterdam University Medical Centre Amsterdam the Netherlands; ^4^ Department of Experimental Vascular Medicine, Amsterdam Cardiovascular Sciences Amsterdam University Medical Centre Amsterdam the Netherlands; ^5^ Animal Research Institute AMC Amsterdam University Medical Centre Amsterdam the Netherlands; ^6^ Department of Pathology, Cardiovascular Research Institute Maastricht (CARIM) Maastricht University Medical Centre Maastricht the Netherlands; ^7^ Dianet Dialysis Centre Amsterdam Amsterdam the Netherlands; ^8^ Department of Nephrology Radboud University Medical Centre Nijmegen the Netherlands; ^9^ Department of Cardiovascular Medicine and Immunology Mayo Clinic Rochester MN; ^10^ Department of Physiology Maastricht University Maastricht the Netherlands

**Keywords:** atherosclerosis, chronic kidney disease, inflammation, kidney replacement therapy, peritoneal dialysis, Atherosclerosis

## Abstract

**Background:**

Atherosclerosis is highly prevalent in people with chronic kidney disease (CKD), including those receiving peritoneal dialysis (PD). Although it is lifesaving, PD induces profound systemic inflammation, which may aggravate atherosclerosis. Therefore, the hypothesis is that this PD‐induced inflammation aggravates atherosclerosis via immune cell activation.

**Methods and Results:**

*ApoE* ^*−/−*^ mice were subjected to a 5/6 nephrectomy to induce CKD. Three weeks later, mice were fed a high‐cholesterol diet. Half of the nephrectomized mice then received daily peritoneal infusions of 3.86% Physioneal for 67 further days (CKD+PD) until the end of the experiment, and were compared with mice without CKD. Sham operated and PD‐only mice were additional controls. CKD+PD mice displayed more severe atherosclerotic disease than control mice. Plaque area increased, and plaques were more advanced with a vulnerable phenotype typified by decreased collagen content and decreased fibrous cap thickness. Increased CD3^+^ T‐cell numbers were present in plaques and perivascular adipose tissue of CKD and CKD+PD mice. Plaques of CKD+PD mice contained more iNOS^+^ immune cells. Spleens of CKD+PD mice showed more CD4^+^ central memory, terminally differentiated type 1 T‐helper (Th1), Th17, and CX3C motif chemokine receptor 1^+^ (CX3CR1) CD4^+^ T‐cells with less regulatory and effector T‐cells.

**Conclusions:**

PD‐fluid exposure in uremic mice potentiates systemic and vascular T‐cell‐driven inflammation and aggravates atherosclerosis. PD polarized CD4^+^ T‐cells toward an inflammatory Th1/Th17 phenotype, and increased CX3CR1^+^ CD4^+^ T‐cells, which are associated with vascular homing in CKD‐associated atherosclerosis. Targeting CD4^+^ T‐cell activation and CX3CR1^+^ polarization has the potential to attenuate atherosclerosis in PD patients.

**Nonstandard Abbreviations and Acronyms**
CX3CR1: CX3C motif chemokine receptor 1
PVAT: perivascular adipose tissue

**Research Perspective**

**What Is New?**

- This study is the first to reveal that peritoneal dialysis fluid exposure in uremic mice specifically aggravates and accelerates atherosclerosis by systemic and vascular T‐cell‐driven inflammation, distinctly to uremia alone.
- Peritoneal dialysis exposure specifically induces increases in T‐lymphocytes in the circulation and the spleen bearing the CX3C motif chemokine receptor 1, which have been associated with vascular homing and atherosclerosis in uremia only.

**What Question Should Be Addressed Next?**

- These data therefore reveal a new therapeutic target for peritoneal dialysis–associated cardiovascular disease, which should be the focus of subsequent studies aiming to inhibit vascular homing.
- This work highlights the eminent and pressing need to reduce the significant pro‐inflammatory side effects of peritoneal dialysis fluids in the clinic by understanding the specific fluid components responsible.

Chronic kidney disease (CKD) is a significant public health concern^1^ that is predicted to rise to the fifth leading cause of years of life lost by 2040.^2^ Patients with continued disease progression toward end‐stage kidney disease are considered for kidney replacement therapies.^3^ Hemodialysis or peritoneal dialysis (PD) are the only options to extend the lives of people with end‐stage kidney disease who are unsuitable for transplantation. PD is widely used; dialysis fluid is infused several times a day into the peritoneal cavity to dwell for a period of time. This causes diffusion of uremic toxins into the fluid across the peritoneal membrane, after which the effluent is drained.^4^

End‐stage kidney disease is a major risk factor for comorbidities,^5^ most notably cardiovascular disease (CVD).^6^, ^7^ In the United States, 52.7% of mortality in PD‐treated people in 2020 was due to CVD, and thus is the leading cause of death in those receiving dialysis.^8^

Atherosclerosis is a key pathological cardiovascular process underlying CVD in CKD. The progression of this chronic inflammatory disease of lipid deposition in the arterial wall^9^, ^10^ develops at an accelerated pace in CKD,^11^ and is already highly prevalent at early stages of CKD.^12^ The mechanisms driving accelerated atherosclerosis in dialysis‐dependent CKD are not fully elucidated.^13^

A unifying mechanism in these phenomena may be inflammation, a prominent feature of CKD.^14^, ^15^ PD‐treated people have particularly high levels of inflammation, likely due partly to the induction of local inflammation in the peritoneal cavity by inherently bio‐incompatible PD fluids.^16^ The high osmotic gradients required for ultrafiltration come from high glucose concentrations (or other osmotic agents),^4^ which may also contribute to untoward metabolic effects.^17^, ^18^ Despite efforts to reduce glucose levels, and the inevitably formed glucose‐degradations products, advanced glycation end products, or use of glucose alternatives, the pro‐inflammatory consequences of PD therapy remain significant.^19^

Inflammation is a lynchpin in atherosclerosis progression, as demonstrated in several studies from our lab,^20^, ^21^, ^22^ and exemplified in the seminal Canakinumab Antiinflammatory Thrombosis in Outcome Study (CANTOS) trial, where anti‐interleukin‐1β therapy markedly reduced adverse cardiovascular events independently of lipid lowering.^23^ A CANTOS substudy demonstrated that inflammation drives residual risk for atherosclerosis specifically in people with CKD, but with no association for serum lipids.^24^ The importance of inflammation extends to the perivascular adipose tissue (PVAT), which is usually atheroprotective^25^ but has deleterious atherogenic influences in high‐fat inflammatory states.^26^, ^27^, ^28^

PD‐treated people are subject to several inflammatory insults resulting from both CKD and PD treatment itself, which may aggravate the accelerated atherosclerotic phenotype in these patients. The aim of this study was to elucidate the effects of CKD and PD on the development and severity of atherosclerosis, specifically if PD‐fluid exposure in addition to CKD (to reflect the clinical setting) adds an additional inflammatory factor and worsens its progression.

For this purpose, we developed and characterized a new PD‐accelerated atherosclerosis model by combining the factors driving atherosclerosis in contemporary clinical PD‐advanced CKD, PD‐fluid exposure, and a high‐cholesterol diet.

## Methods

### Data Access and Responsibility

The data, analyses, and summary statistics supporting the findings of this study are available from the corresponding author upon reasonable request.

### Animal Experiments

In‐house *ApoE* ^*−/−*^ mice were bred and housed according to institutional guidelines. Animal experiments were approved by the local ethics committees (VU Medical Centre and Academic Medical Centres, Amsterdam, the Netherlands) and the Dutch National Central Committee on animal experimentation (AVD11400020209344), in conformation with directive 2010/63/EU of the European Parliament and the Animal Research: reporting of in vivo experiments (ARRIVE) guidelines.

### Induction of CKD

Twelve‐week‐old male and female mice were subjected to 5/6 nephrectomy as previously described.^29^ In brief, mice were placed under isoflurane anesthesia (1 L/min O_2_ and air, 3.5% to 4% induction, 2% to 3% maintenance) after receiving preoperative analgesia (0.05 mg/kg s.c. buprenorphine; Schering‐Plough, Kenilworth, NJ). An abdominal midline incision was made and the capsule of the left kidney was removed. The upper and lower lobes were then cut away and cauterized (High temperature fine tip cautery pen [Bovie Medical, Clearwater, FL; #AA01]). Next, the right kidney was decapsulated followed by total ligation of the renal blood vessels and ureter, and the right kidney was removed. Soluble sutures were applied to the muscle and skin layers. Mice received twice daily injections of 0.05 mg/kg buprenorphine for 2 days following surgery. Sham surgeries included decapsulation of both kidneys and analgesic treatment but no kidney tissue was removed.

### Peritoneal Dialysis Infusions

Fourteen days later, half of the nephrectomized, and PD‐only animals, had peritoneal access ports installed to facilitate peritoneal dialysis fluid exposure, as previously described^30^ (MousePort with 4 French catheter [UNO Roestvaststaal BV, Zevenaar, the Netherlands; #MMP‐4S]).

One week later, once‐daily infusions of 2 mL of body temperature Physioneal (3.86% [Baxter Healthcare Ltd, Thetford, UK]) were started via the port using specialized needles (PosiGrip HuberPoint Needle, 25 ga × 1/2″ [UNO Roestvaststaal BV, Zevenaar, the Netherlands; #PG25‐500]) and continued for 67 consecutive days. At the same time as the port installations, all groups of mice were placed on a high‐cholesterol (0.15%) diet (Altromin, Lage, Germany; #C1000 mod) for 9 weeks until the end of the experimental period. The PD‐only group included exposure to PD fluids, a sham surgery, and the high‐cholesterol diet only.

The dropout rate was 12.1% as shown in Table S1. Weight loss following surgery was similar between male and female mice (Figure S1A), whereas females recovered faster (Figure S1B).

### Histology

Mice were terminated via exsanguination and blood was collected via right ventricular puncture with EDTA‐coated syringes. Ice‐cold phosphate‐buffered saline was then perfused via the left ventricle. The arterial tree was fixed in 1% paraformaldehyde, while other tissues were fixed in 4% paraformaldehyde. All tissues were dehydrated, embedded in paraffin, and sectioned at 4 μm. Chemical stains performed included hematoxylin and eosin and picrosirius red.

Materials used are listed in Tables S2–S7, and immunohistochemical stains were performed as previously described.^22^ In brief, tissue sections were rehydrated in xylene and 100% ethanol treatment. Sections were treated with 0.3% H_2_O_2_ in absolute methanol, and sequentially with 100%, 96%, 70%, and 50% ethanol. Tissues were treated with the appropriate antigen retrieval buffer at 95 °C. Antibodies, antibody detection kits, and chromogens were applied at room temperature. Finally, slides were counterstained in hematoxylin. Where applicable, reagents were diluted in Tris‐buffered Tween (Tris‐buffered saline, 1% bovine serum albumin, 0.1% Tween).

### Microscopy and Image Analysis

Images were acquired using an Olympus Slideview VS200 slide scanner (Olympus Corporation; Tokyo, Japan). Aortic arch sections were stained with hematoxylin and eosin and were examined for a 100‐μm range in which the arch and its bifurcations were open and the most plaque was visible; all further analyses were performed within this range. Plaque readouts were obtained using QuPath image analysis software v0.3.2 (Open‐source software,^31^ Peter Bankhead). Plaque staging was qualitatively assessed using the Virmani classification^32^ by a blinded single operator. Liver and intestinal pathology was also assessed by a blinded single operator. For plaque analyses, only female mice were used because the male group was of insufficient size for statistical comparisons.

### Serum Cholesterol and Triglyceride Measurements

Serum concentrations of total cholesterol and triglycerides were assessed using the CHOD/GPO‐PAP method kits (BioLabo; Maizy, France; #87356 and #87319, respectively) according to manufacturer's instructions. Very‐low‐density lipoprotein (VLDL), low‐density lipoprotein (LDL), and high‐density lipoprotein (HDL) subfractions of cholesterol were analyzed using fast‐protein liquid chromatography as previously described.^33^

Hepatic concentrations of triglycerides and cholesterol were measured using commercially available kits from DiaSys (Holzheim, Germany) following lipid extraction from snap frozen liver tissue according to Bligh and Dyer.^34^

### Flow Cytometry

Blood, spleen, and mesenteric lymph nodes were collected. Blood and spleen were exposed to red blood cell lysis buffer (1.29 mmol EDTA, 10 mmol NaHCO_3_, and 157 mmol NH_4_Cl in milliQ water). Cells were then stained in flow cytometry buffer (2.53 mmol EDTA and 5 g/L BSA in phosphate‐buffered saline, pH set 7.4–7.8) with extracellular antibodies. For intracellular stains, the cells were also fixed and permeabilized in Fix/Perm buffer (Thermo‐Fisher; Waltham, MA; #5523). Data acquisition was performed on either FACS Canto II B, LSR Fortessa, or FACS Symphony A1 machines where appropriate (all BD Biosciences; Franklin Lakes, NJ).

All antibodies and dilutions (listed in Tables S2–S7) were used extracellularly except where noted. Data analyses were performed using FCSExpress software v7 (DeNovo Software; Pasadena, CA). All gating strategies are shown in Figures S2, S3, and S4.

### Statistical Analysis

Quantitative results were analyzed firstly with the ROUT method outliers test (Q=1%), and then using a 1‐way ANOVA with Tukey's multiple comparison test except where noted. A *P* value of <0.05 was considered statistically significant. Data are presented as means±SD except where noted. All statistical analyses were performed using GraphPad Prism v9.3.1 (GraphPad Software Ltd; Boston, MA).

## Results

### 5/6 Nephrectomy Induces Kidney Failure

Serum urea and creatinine concentrations were measured to verify that the surgical interventions were effective in inducing kidney failure, and to rule out an independent effect of the surgery itself.

**Figure 1:**
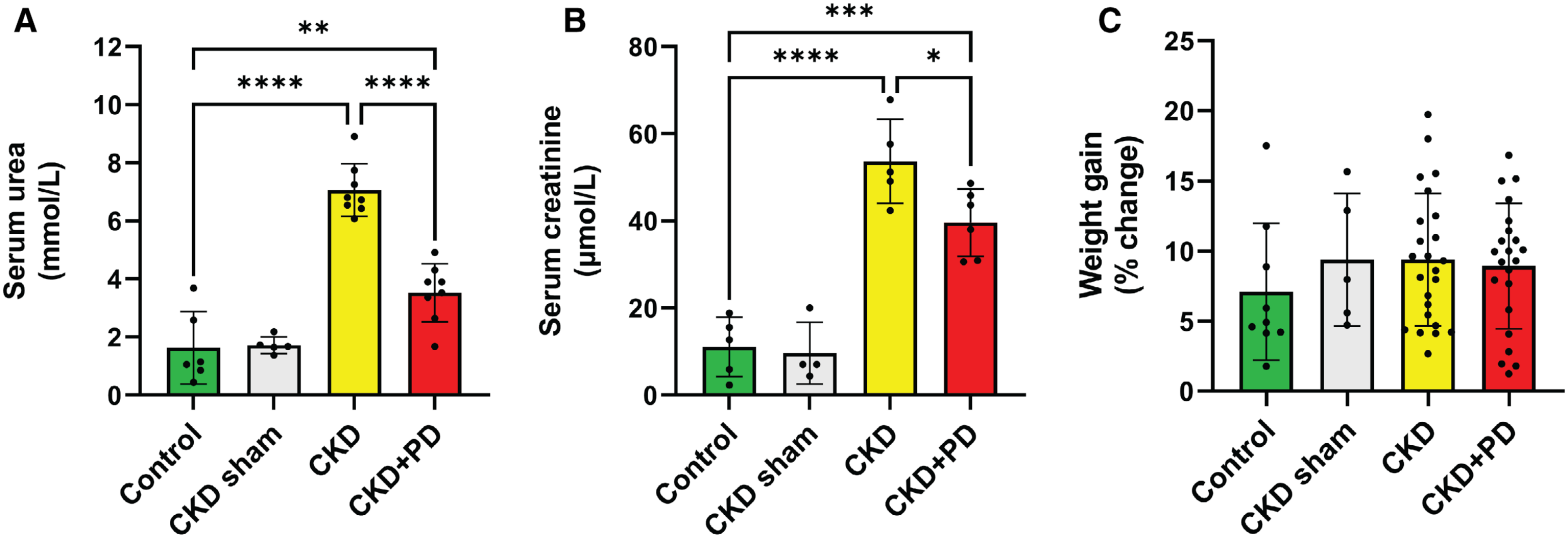
Serum urea and creatinine concentrations increase following 5/6 nephrectomy while body weight is unchanged (**A**) Serum urea concentrations (n=8 for control, CKD, and CKD+PD. n=5 for CKD sham). (**B**) Serum creatinine concentrations (n=8 for control, CKD, and CKD+PD. n=5 for CKD sham). (**C**) Change in body weight expressed as percentage change over the course of the 9‐wk high‐cholesterol diet period in control (n=9), CKD sham (n=5), CKD (n=24), and CKD+PD (n=22) conditions, respectively. Bars represent means with SD. *P ≤0.05; **P ≤0.01; ***P ≤0.001; ****P ≤0.0001. CKD indicates chronic kidney disease; and PD, peritoneal dialysis.

Serum urea and serum creatinine concentrations were substantially increased in the CKD and CKD+PD groups versus the control and sham groups; both measures were also different between CKD and CKD+PD groups (Figure 1A and 1B). Percent body weight increase between groups across the 9‐week diet period was similar in all groups (Figure 1C).

### Combining Kidney Failure and Peritoneal Dialysis Worsens Atherosclerotic Burden

**Figure 2:**
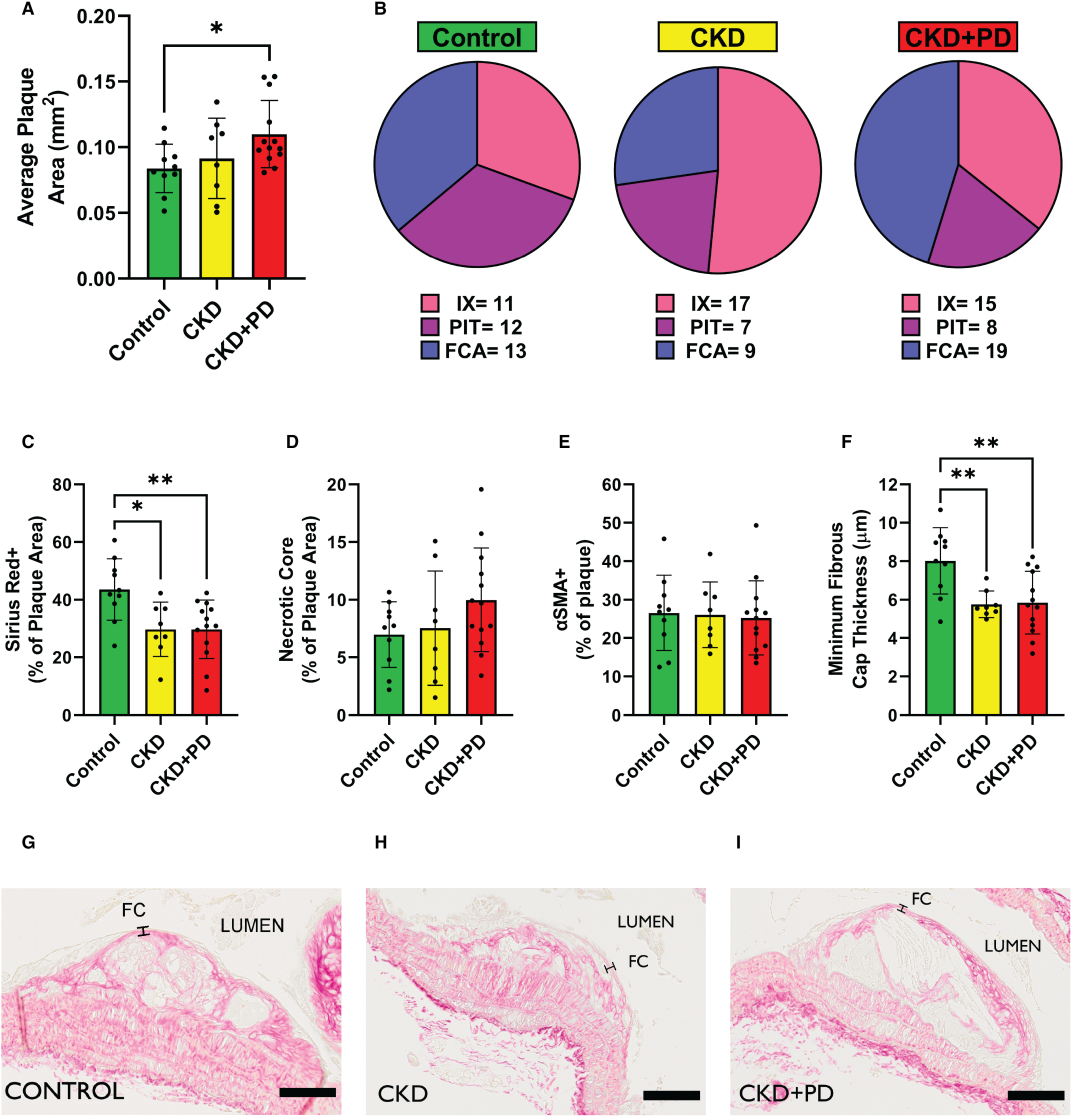
Chronic kidney disease plus peritoneal dialysis increases atherosclerotic plaque area and reduces plaque stability Plaque analyses are shown in female *ApoE* ^*−/−*^ mice in control, CKD, and CKD+PD conditions. (**A**) Mean plaque area in the aortic arch with SD. (**B**) Qualitative categorization of plaque severity via Virmani classification shown as number of total plaques measured in each group; FCA indicates fibrous cap atheroma (blue); IX, intimal xanthoma (pink); and PIT, pathological intimal thickening (purple). (**C**) Mean percentage plaque area positive for Sirius red staining with SD. (**D**) Mean percentage of plaque area containing necrotic core with SD. (**E**) Mean percentage plaque area positive for α‐smooth muscle actin (αSMA) with SD. (**F**) Mean minimum fibrous cap thickness in each mouse plaque with SD. (**G–I**) Representative images of plaque in control (**G**), CKD (**H**), and CKD+PD (**I**) conditions. n=13 for all plaque measurements. CKD indicates chronic kidney disease; FC, fibrous cap; and PD, peritoneal dialysis. *P ≤0.05; **P ≤0.01. Scale bars represent 100 μm.

Atherosclerosis burden was assessed in female *ApoE* ^*−/−*^ mice with CKD and mice with CKD and PD‐fluid exposure and compared with control mice. Plaque area in the aortic arch was similar in CKD mice but was increased in CKD+PD mice compared with control (Figure 2A, 2G through 2I). Plaque area was similar to the control group in CKD sham (surgery with no kidney tissue removed) mice and also in PD‐only mice (PD fluid exposure in mice with normal kidney function) (Figure S5A). CKD mice displayed attenuated advancement of atherosclerosis compared with control with more intimal xanthomas, less pathological intimal thickenings, and less fibrous cap atheromas. In contrast, CKD+PD mice had an increased number of plaques and accelerated plaque advancement as reflected by more fibrous cap atheromas compared with control mice.

Additionally, collagen content, quantified as Sirius red positivity (Figure 2C), was decreased in CKD and CKD+PD groups versus control. Necrotic core size was similar in all groups (Figure 2D), as was α‐smooth muscle actin content (Figure 2E). However, minimum fibrous cap thickness was decreased in both CKD and CKD+PD groups versus control (Figure 2F through 2I).

Taken together, these data show that CKD induces a vulnerable plaque phenotype, but that plaque size is only increased when CKD and PD are combined and CKD‐associated atherosclerotic burden is aggravated. CKD+PD conditions increase plaque number and size, induce more advanced plaques and a vulnerable plaque phenotype.

### Combining CKD and PD‐Fluid Exposure Enhances T‐Cell and Macrophage Vascular Inflammation

**Figure 3:**
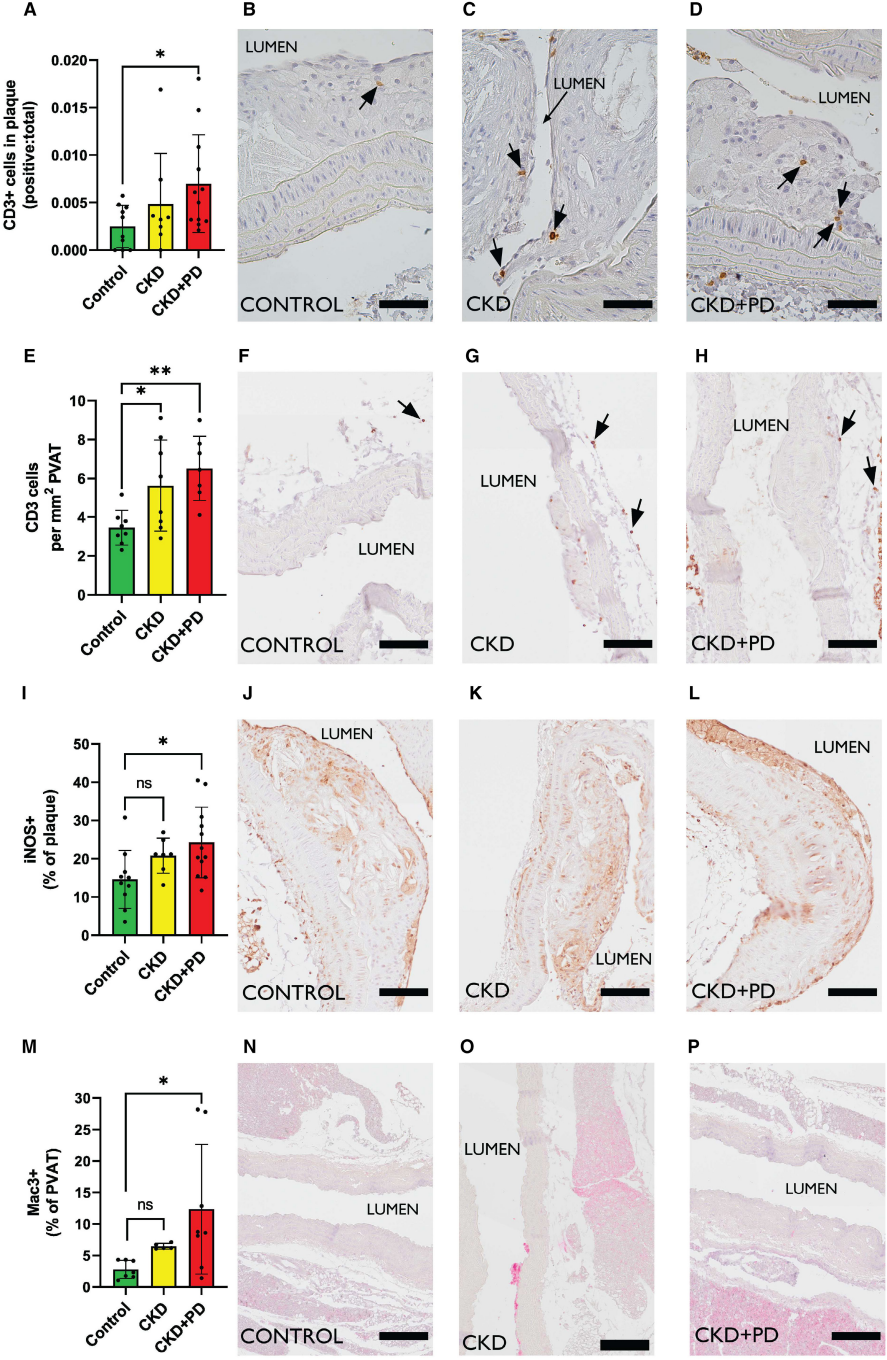
Chronic kidney disease and peritoneal dialysis in combination induces immune cell infiltration into aortic arch plaques and surrounding perivascular adipose tissue (PVAT) Immune cell positivity in the aortic arch plaques and PVAT of *ApoE* ^*−/−*^ mice under control, induced CKD, and induced CKD plus peritoneal dialysis exposure conditions are shown. (**A**) Quantity of CD3^+^ cells present in the plaque indexed against total cell count of each plaque with representative images with arrows indicating positive cells (**B–D**). (**E**) Quantity of CD3^+^ cells present in the PVAT indexed against area with representative images with arrows indicating positive cells (**F–H**). (**I**) Percentage of the plaque positive for iNOS with representative images (**J–L**). (**M**) Percentage of PVAT positive for Mac3 with representative images (**N–P**). *P ≤0.05; **P ≤0.01. Scale bars represent 100 μm. CKD indicates chronic kidney disease; and PD, peritoneal dialysis.

Next, the immune cell composition of plaques and PVAT was assessed to capture local atherogenic effects potentiated by PVAT.^26^ T‐cells in the plaque were increased in the CKD+PD group versus control (Figure 3A through 3D), whereas both CKD and CKD+PD groups showed more T‐cells in PVAT (Figure 3E through 3H).

While the overall macrophage content of CKD and CKD+PD plaques were unchanged versus control (Figure S5B), there was an increase in pro‐inflammatory iNOS positivity in the CKD+PD group (Figure 3I through 3L), which is prominently produced by M1 macrophages. This suggests a shift of plaque macrophages to a pro‐inflammatory phenotype. Macrophage content in the PVAT was increased in the CKD+PD group, but not CKD group versus control (Figure 3M through 3P), indicating that inflammation in the environment around the plaque is important for plaque severity.

In summary, CKD+PD aggravated arterial inflammation by inducing infiltration of T‐cells and pro‐inflammatory iNOS+ cells into atherosclerotic plaques and PVAT, whereas CKD conditions induced only influxes of T‐cells into PVAT.

### CKD and PD‐Fluid Exposure Induce a Pro‐Inflammatory Systemic T‐Cell Response in the Blood and Spleen

To assess the systemic and local immune response to CKD and PD‐fluid exposure, the spleen, whole blood, and mesenteric lymph nodes were collected and analyzed using flow cytometry for immune cell markers.

**Figure 4:**
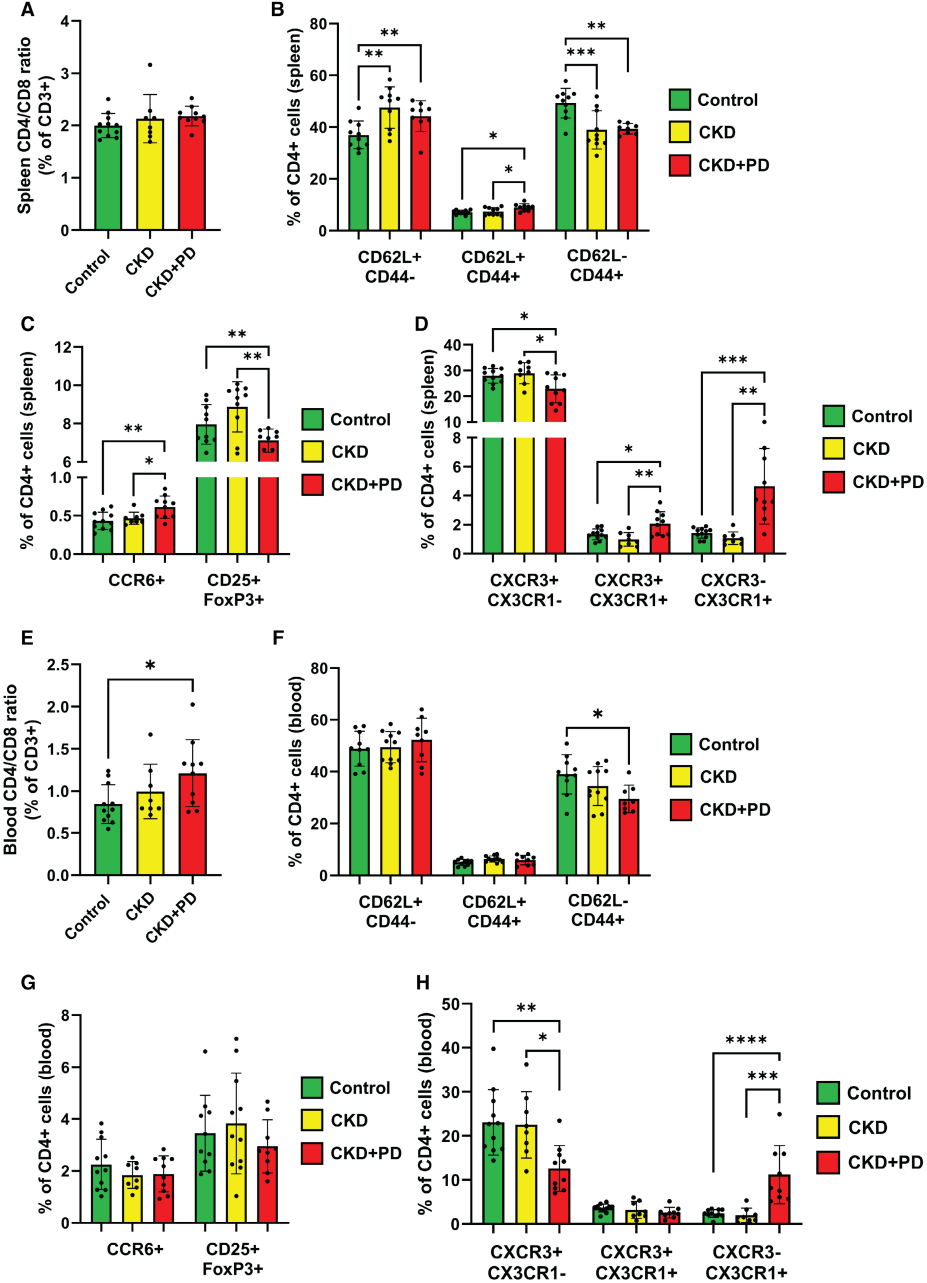
Chronic kidney disease and peritoneal dialysis induce systemic immune changes in T‐cell subsets Percentage of cells of each population containing stated T‐cell markers are shown in control, CKD, and CKD+PD groups (n=10 for each). (**A**) CD4/CD8 ratio of CD3^+^ cells in the spleen. (**B**) Activation state of CD4^+^ cells in the spleen. (**C**) Relative levels of CD4^+^ subsets in the spleen. (**D**) Relative levels of CD4^+^ cells in the CXCR3/CX3CR1 axis in the spleen. (**E**) CD4/CD8 ratio of CD3^+^ cells in the blood. (**F**) Activation state of CD4^+^ cells in the blood. (**G**) Relative levels of CD4^+^ subsets in the blood. (**H**) Relative levels of CD4^+^ cells in the CXCR3/CX3CR1 axis in the blood. Markers used include CD62L^+^/CD44^−^ (naive), CD62L^+^/CD44^+^ (memory), CD62L^−^/CD44^+^ (effector), CCR6^+^ (Th17), CD25^+^/FoxP3^+^ (regulatory T‐cells), CXCR3^+^/CX3CR1^−^ (effector Th1), CXCR3^+^/CX3CR1^+^ (terminally differentiated Th1), and CXCR3^−^/CX3CR1^+^ (vascular homing). *P ≤0.05; **P ≤0.01; ***P ≤0.001; ****P ≤0.0001. CKD indicates chronic kidney disease; and PD, peritoneal dialysis.

In the spleen, the CD4/CD8 ratio was similar between all groups (Figure 4A). There was a higher percentage of naive CD4^+^ cells (CD62L^+^/CD44^−^), a lower percentage of effector CD4^+^ cells (CD62L^−^/CD44^+^) in CKD and CKD+PD groups, and a higher percentage of central memory CD4^+^ cells (CD62L^+^/CD44^+^) in the CKD+PD group versus control (Figure 4B). Splenic CD8^+^ cell changes were milder, naive CD8^+^ and central memory CD8^+^ cells were similar between all groups, whereas CD8^+^ effector cells decreased (Figure S6A).

There were several changes in splenic CD4^+^ T‐cell subtypes. There was a higher percentage of Th17 cells (CCR6^+^), and a lower percentage of regulatory T‐cells (CD25^+^/FoxP3^+^) in CKD+PD mice as compared with control (Figure 4C). There was a lower percentage of effector Th1 cells (CXCR3^+^/CX3CR1^−^) and a higher percentage of terminally differentiated Th1 cells (CXCR3^+^/CX3CR1^+^) and vascular homing cells (CXCR3^−^/CX3CR1^+^) in CKD+PD groups versus control (Figure 4D). Th2 (CD4^+^/CCR4^+^) and follicular T‐helper (CD4^+^/PD‐1^+^/CXCR5^+^) cells were unchanged in CKD+PD conditions, whereas in CKD conditions there was a decrease in Th2 cells versus controls (Figure S6B).

In the blood, the ratio of CD4 to CD8 cells increased in CKD+PD groups compared with control (Figure 4E). Naive CD4^+^ and central memory CD4^+^ cells were similar between groups, whereas there was a lower percentage of effector CD4^+^ cells in CKD+PD mice as compared with control (Figure 4F). Naive, central memory, and effector CD8^+^ cells were similar between all groups (Figure S6C). Among blood CD4^+^ cells, there were similar percentages of Th17 cells and regulatory T‐cells (Figure 4G). There was a lower percentage of effector Th1 cells, similar levels of terminally differentiated Th1 cells, and a higher percentage of vascular homing cells in the CKD+PD group versus control (Figure 4H). The percentages of Th2 and follicular T‐helper cells were similar in all groups (Figure S6D). Notably, all the aforementioned cellular population changes in Figure 4 were absent in the mesenteric lymph node (Figure S7A and S7B).

In the splenic myeloid population, there was a lower percentage of overall monocytes (CD11b^+^/Ly6G^−^) and patrolling monocytes (Ly6C^low^), with a higher percentage of pro‐inflammatory monocytes (Ly6C^high^) in CKD+PD conditions versus control (Figure S8A). Additionally, there was a lower percentage of immature (CD11c^+^/MHCII^−^) and mature (CD11c^+^/MHCII^+^) dendritic cells in CKD+PD groups versus control (Figure S8B).

There was a lower percentage of monocytes, and a higher percentage of pro‐inflammatory monocytes in the blood in CKD+PD conditions versus control (Figure S8C). There was also a lower percentage of immature dendritic cells in CKD+PD conditions, whereas the percentage of macrophages (F4/80^+^) increased in CKD conditions but decreased in CKD+PD, all versus control (Figure S8D).

Among splenic B‐cells there were more follicular B2 (CD23^+^) and less marginal zone (CD23^−^/IgM^+^) cells as a percentage of total B2 cells (CD19^+^/B220^+^) in CKD conditions compared with control (Figure S9A). Similarly, in blood there were more follicular B2 and less marginal zone cells in CKD+PD conditions. Finally, we observed less plasma cells (CD19^−^/B220^−^/CD138^+^) only in the CKD group as compared with control (Figure S9B).

In summary, these data show that CKD+PD reshapes the systemic immune response, predominantly in CD4^+^ T‐cell populations, toward a more chronically activated Th1‐like inflammatory response.

### Altered Lipid Handling and Hepatic Inflammation Is Present in CKD and CKD+PD Mice

**Figure 5:**
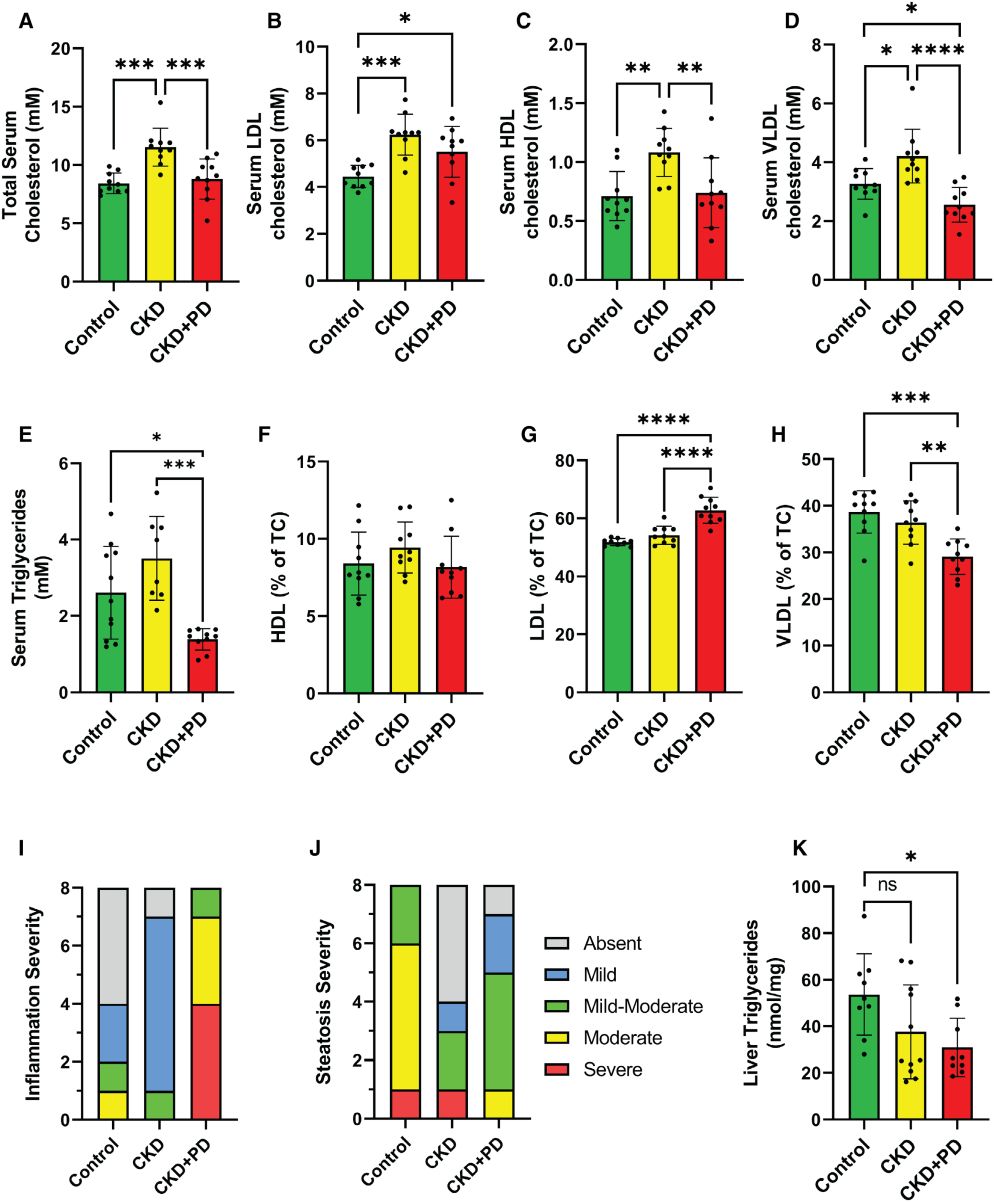
Dyslipidemia and hepatic inflammation are present following chronic kidney disease and PD‐fluid exposure Serum concentrations in *ApoE* ^*−/−*^ mice under control, CKD, and CKD plus PD conditions are shown along with liver histology. Data are expressed as means±SD, except for (**I**) and (**J**). (**A**) Total serum cholesterol concentrations, and constituent subfractions, (**B**) high‐density lipoprotein (HDL), (**C**) low‐density lipoprotein (LDL), and (**D**) very low‐density lipoproteins (VLDL). The ratio of these subfractions is shown as a percentage of the total cholesterol concentration for (**F**) HDL, (**G**) LDL, and (**H**) VLDL. (**E**) Total serum triglyceride concentrations. Assessment of liver pathology in (**I**) qualitative assessment of inflammation and (**J**) steatosis is shown with different categories of severity for each group. (**K**) Concentration of triglycerides in liver tissue. *P ≤0.05; **P ≤0.01; ***P ≤0.001; ****P ≤0.0001. n=10 for all. CKD indicates chronic kidney disease; and PD, peritoneal dialysis.

Sera and liver samples were collected to assess lipoprotein metabolism. In CKD mice, total serum cholesterol, absolute levels of HDL, LDL, and VLDL cholesterol were higher versus control mice (Figure 5A through 5D). Considering LDL and VLDL cholesterol subfractions are more atherogenic than HDL,^35^ their relative distribution was assessed. The ratio of HDL, LDL, and VLDL cholesterol fractions were similar in CKD groups as compared with control (Figure 5F through 5H).

CKD+PD mice showed higher LDL and lower VLDL cholesterol concentrations in absolute and relative terms compared with control, whereas HDL and total cholesterol concentrations were similar (Figure 5A through 5D, 5F through 5H). Serum triglyceride concentrations were lower in the CKD+PD group versus controls (Figure 5E).

To assess whether inflammation induced by PD‐fluid exposure damaged the intestine and impaired cholesterol handling, pathological assessment of the digestive tract was performed. CKD and CKD+PD conditions did not alter villi structure in the stomach, intestine, or colon as compared with control (Table S8). In CKD+PD mice, hepatic inflammation increased versus other groups (Figure 5I). Hepatic steatosis was similar in CKD and CKD+PD groups versus control (Figure 5J). Finally, CKD+PD mice had lower liver triglyceride concentrations (Figure 5K), whereas liver collagen and cholesterol levels were similar (Figure S10).

Taken together, these data demonstrate that CKD mice have higher absolute, but not relative, quantities of total cholesterol and all cholesterol subfractions versus control mice. In contrast, CKD+PD mice have higher LDL and lower VLDL levels in absolute and relative terms. Additionally, CKD+PD mice expressed more severe liver inflammation and lower liver triglyceride concentrations. PD‐induced hepatic inflammation in this context may not be associated with lipoprotein levels, as CKD+PD mice had the most severe liver inflammation without lipoprotein changes comparable to CKD mice.

## Discussion

In this study we show that PD exposure in uremic mice accelerates atherosclerosis by inducing systemic inflammation resulting in larger, more advanced, more vulnerable plaques, and accelerated new plaque formation. Compared with controls, CKD itself resulted in a trend toward increased plaque burden and increased plaque inflammation. However, when CKD mice were also subjected to PD, plaque burden, as well as plaque inflammation and vulnerability, increased significantly compared with controls. Plaque changes in CKD+PD mice are likely driven by T‐cell and inflammatory cell infiltration into the plaque and PVAT. In turn, CKD mice had a milder phenotype than mice also exposed to PD, having only more vulnerable plaques compared with control. The addition of PD created a unique plaque phenotype distinct from that induced by CKD only.

The T‐cell response in the spleen and blood of CKD+PD mice was markedly changed, with chronically activated T‐cells, shown as more central memory and terminally differentiated Th1 cells. CKD+PD mice had more vascular homing (CX3CR1^+^) T‐cells, a subtype reported to target and infiltrate the aortic wall to aggravate atherosclerosis.^36^ Concomitantly, CKD+PD mice showed less effector and regulatory T‐cells, suggesting a reduced ability to resolve inflammation or respond to new threats. CKD mice showed a milder response with only increased naive CD4^+^ cells and decreased CD4^+^ effectors in spleen and blood.

PD exposure in uremic mice undoubtedly triggers local peritoneal inflammation,^37^ but here we show a strong CD4^+^ T‐cell and macrophage phenotype distant to the peritoneal cavity, leading to aggravated atherosclerosis. Strikingly, all of these changes were absent in the mesenteric lymph node, the first site of peritoneal drainage,^38^ suggesting PD‐induced systemic inflammation is not necessarily caused by local peritoneal inflammation, or may bypass the local lymphatic drainage system.

### Effects of PD on Atherosclerosis and Immunity

To our knowledge this is the first direct evidence in any context that exposure to PD fluids accelerates atherosclerosis and causes larger more advanced plaques. In PD‐treated people, no data exist that describe specific plaque characteristics. As our data now reveal, the specific context of PD uniquely shapes murine atherosclerotic development.

In our study, CKD mice showed plaque vulnerability but no increases in lesion size. Similar studies in nephrectomized *ApoE* ^*−/−*^ mice show a plaque phenotype of vulnerability^39^ or increased lesion size,^40^, ^41^, ^42^, ^43^, ^44^ and usually the latter (rarely both). Different protocols, diets, strains, techniques, and facilities likely underpin the variable results but taken together, the plurality of evidence suggests that a uremia‐only setting is insufficient to induce both a vulnerable and larger plaque phenotype in *ApoE* ^*−/−*^ mice. Bi et al.^45^ were the notable exception, as they reported both increases in lesion size and necrotic core, and partially in agreement with our findings, reduced vascular smooth muscle cell content and decreased fibrous cap area.

Plaque pathology from our CKD mice, and most of the aforementioned studies, are in line with CKD patient data, which show increased plaque severity and advanced stage,^46^, ^47^ plaque inflammation,^48^ and vulnerability without lesion size changes^12^, ^49^, ^50^, ^51^ in advanced stages of CKD.^52^ The present work provides compelling evidence that PD specifically worsens plaque burden beyond uremia only, which should be confirmed in patients.

Systemically, the chronic T‐cell activation phenotype reported here is well supported by other data sets from uremic mice^53^, ^54^ and people with CKD^55^, ^56^ who show increased T‐cell differentiation with more memory T cells and fewer naive T cells. We describe a substantial increase in CX3CR1^+^ CD4^+^ T cells, similarly to Dong et al., who demonstrated increased lesion size, Th17 polarization, and promotion of aortic CX3CR1^+^ T‐cell accumulation after adoptive transfer of CX3CR1^+^ T‐cells into nephrectomized CX3CR1^−/−^ mice.^36^ Additionally, our CKD+PD mice had fewer regulatory T cells and more Th17 cells, similarly to CKD^57^ and dialysis patients,^58^ where the degree of change in Th17 and regulatory T‐cell number correlates strongly with CKD severity.^59^, ^60^ These T‐cell changes in turn can worsen atherosclerotic burden.^61^, ^62^ Future studies should seek to understand which specific factors of PD‐fluid exposure are driving the increases in CX3CR1^+^ T cells, such as inflammatory glucose degradation products or advanced glycation end products in PD fluids.^63^

Macrophages and T cells accumulated in the PVAT of CKD+PD mice, which is a firmly established contributor to worsened atherosclerosis outside of the adipose tissue.^27^ Although this is the first report of PVAT involvement in atherosclerosis secondary to CKD+PD, Kawahito^64^ and colleagues previously showed that PVAT contributes to atherosclerosis in uninephrectomized *ApoE* ^*−/−*^ mice by activation of the renin‐angiotensin system. Moreover, Du et al. recently demonstrated a Th1 and regulatory T‐cell imbalance in a model of bacteria‐induced chronic inflammation,^65^ in which PVAT inflammation preceded endothelial inflammation and triggered atherosclerosis. The temporal sequence in atherosclerosis development remains contested and is beyond the scope of this study, but a Th1 and regulatory T‐cell imbalance and PVAT involvement in atherosclerosis are in agreement with our data.

Lipid analysis showed higher absolute cholesterol levels with no triglyceride changes in CKD mice. In CKD+PD mice, the ratio of subfractions was changed: more LDL and less VLDL cholesterol, with triglyceride decreases in liver and serum. Previous studies also show higher cholesterol following nephrectomy or chemical induction of CKD in *ApoE* ^*−/−*^ mice.^40^, ^66^, ^67^, ^68^ The cholesterol changes in our CKD mice resembles the clinical phenotype. Dyslipidemia in people with CKD is characterized by increased total, LDL, and non‐HDL cholesterol with decreases in HDL cholesterol.^69^ PD patients may be broadly similar but the direct effects remain unclear at best.^70^, ^71^, ^72^, ^73^ In CKD+PD mice there were no absolute cholesterol changes as in CKD mice but increased hepatic inflammation, yet those mice had the worst phenotype. Given that lipid levels are not associated with increased cardiovascular risk ratio in patients with CKD,^24^ and the reported inefficacy of statin therapy in PD‐patient groups,^74^, ^75^ lipid changes may be much less important here than under normal kidney function conditions.

### Strengths and Limitations

This study utilized a novel operational model comprising physical inductions of CKD and peritoneal inflammation that could be easily transferred to other lab contexts, and may reasonably reflect human clinical conditions. The *ApoE* ^*−/−*^ line is also a very well‐studied model of atherosclerosis.^76^ The procedures were well tolerated, with minimal dropout, and with kidney dysfunction comparable to previous studies in our lab.^77^ However, the nephrectomy surgery induces advanced CKD and not end‐stage kidney disease because the mouse is not dialysis dependent. Relatedly, mouse PD‐fluid infusions are not a kidney replacement therapy. Although human exposure to PD fluid has a much longer duration, our deliberate choice of fluid with the highest glucose and therefore highest glucose degradation product (GDP) concentrations with a long‐term exposure recapitulates well the deleterious effects of human PD‐therapy. We did not focus on which component of the PD fluid was the culprit for inducing the observed immune responses, but hyperglycemia may be a contributor to inflammation. Protein glycation has been implicated in the development of macrovascular disease in some studies,^78^, ^79^ but in the Maastricht cohort this was only true for microvascular disease.^80^

We describe an advanced stage of atherosclerosis; however, the progression and development at earlier time points would provide a more granular picture. Similarly, while we see strong T‐cell responses, this is likely the matured phenotype where immune dysfunction has begun and earlier time points may illuminate the development of systemic inflammation. Plaque data were only from female mice, since the male group size was too low for statistical analysis. It is probable that male mice would have had the same plaque phenotype, but we cannot confirm that here. Finally, CKD‐ and PD‐related CVD is not limited to atherosclerosis. Advanced CKD nonatherosclerotic CVD, such as medial calcification, is at least as dominant as atherosclerotic disease,^6^ but here we considered our model not representative for that aspect of CVD in CKD and dialysis.

### Future Clinical Implications

We clearly show that chronic exposure to PD fluid is an accelerating factor in atherosclerosis development in mice, and this is likely to be true in PD patients. Implementing improvements to PD fluids that ease systemic inflammation in mice such as lithium chloride^81^ or steviol glycosides^37^ may therefore attenuate atherosclerotic disease in patients secondary to PD fluids. More generally, the present model is a new tool to test these and other therapeutic avenues. Clinically, the substantial increases in CX3CR1^+^/CD4^+^ T‐cells observed here are of particular note since inhibition of CX3CR1 to ameliorate atherosclerosis progression is promising,^82^, ^83^ as is CX3CR1 modulation in the treatment of CKD.^84^ These therapeutic targets may therefore be effective in treating CKD+PD–related atherosclerotic disease.

## Conclusions

**Figure 6:**
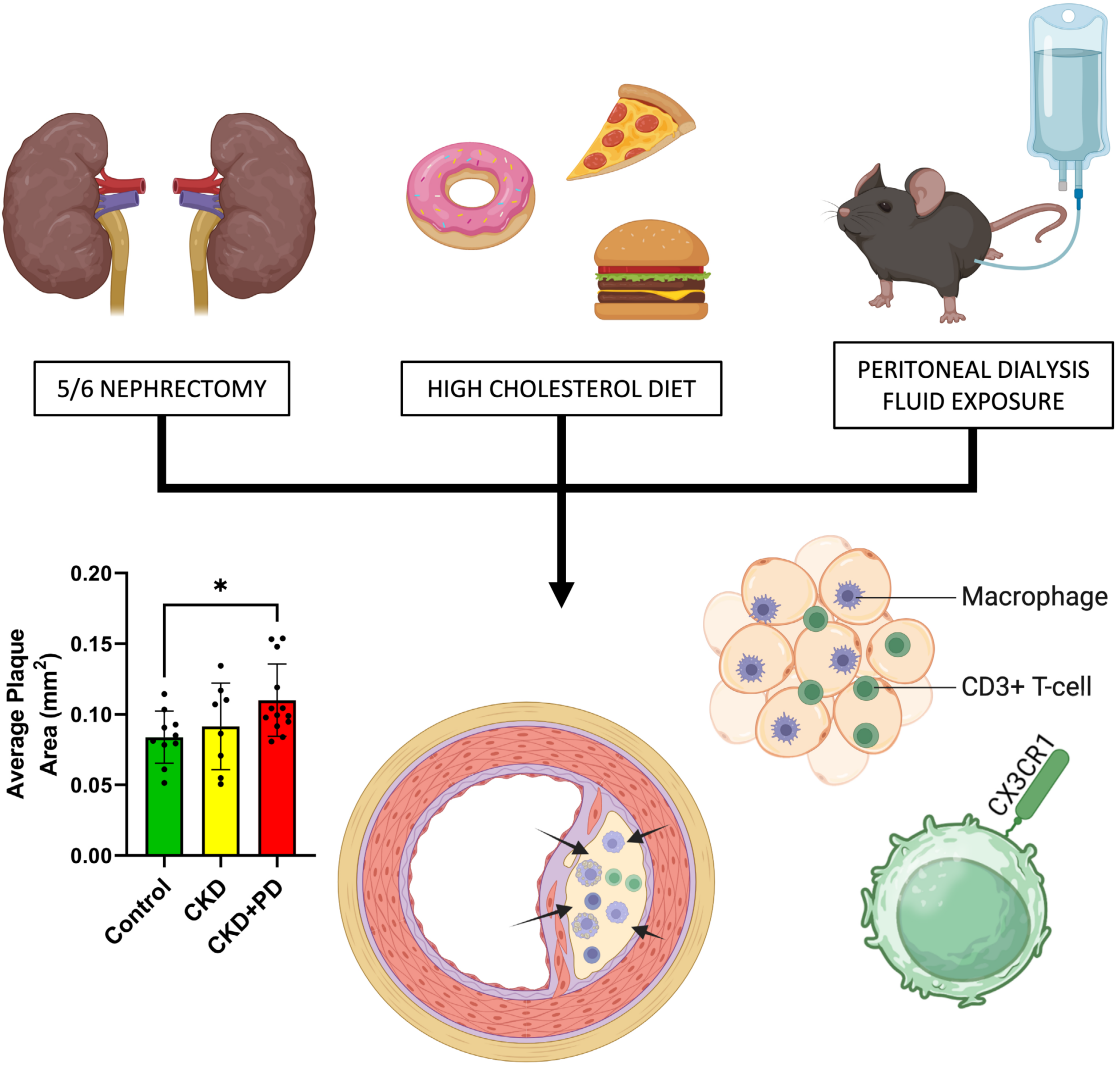
Peritoneal dialysis–fluid exposure in uremic mice accelerates and aggravates atherosclerosis CKD+PD synergistically aggravates atherosclerosis via T‐cell‐ and macrophage‐driven intraplaque and perivascular adipose tissue inflammation. Additionally, CKD+PD drives systemic CD4^+^ T‐cell changes, while CX3C motif chemokine receptor 1^+^ T‐cells may be a new therapeutic avenue. Figure created with BioRender. *P ≤0.05. CKD indicates chronic kidney disease; and PD, peritoneal dialysis.

To conclude, we demonstrate that PD in a CKD setting aggravates atherosclerotic disease, leading to larger and more vulnerable plaques. This is likely driven by a profound remodeling of the systemic CD4^+^ T‐cell response, with a notable increase in the percentage of CD4^+^ T‐cells expressing the vascular homing receptor CX3CR1, which may shed new light on the causative factors. Furthermore, PD induces rampant vascular inflammation in the perivascular adipose tissue and plaques by T cells and macrophages to drive disease development. These changes are brought together and shown graphically in an overall summary in Figure 6. This study uncovers important new influences of PD fluids and PD therapy on atherosclerotic disease in a CKD context.

## Sources of Funding

This project has received funding from the European Union's Horizon 2020 research and innovation program under the Marie Skłodowska‐Curie Actions, grant agreement No. 812699 (to J.K). E.C.E. is supported by the Dutch Heart Foundation (grant number 2020B008).

## Disclosures

None.

## Supporting information

Tables S1–S8
Figures S1–S10
