# Supplementary material for: Peritoneal Dialysis Aggravates and Accelerates Atherosclerosis in Uremic ApoE −/− Mice
Source: J Am Heart Assoc. 2024 Jul 9;13(14):e034066. doi: 10.1161/JAHA.123.034066 (PMC11292770; doi:10.1161/JAHA.123.034066)
Supplement: Supplementary file 1 — Tables S1–S8 Figures S1–S10 [file JAH3-13-e034066-s001.pdf]

SUPPLEMENTAL MATERIAL

Table S1: Most dropouts in the study were in male mice.

	Male	Female	Total
**Surgery related dropouts**	4	0	4
**Other dropouts**	5	3	8
			12/99

Surgery related dropouts were defined as

any complication necessitating a humane end point in the immediate 7-day recovery phase following the 5/6 nephrectomy, whilst other dropouts were any other reason outside of this time period.

Flow cytometry antibodies

Name	Manufacturer	Dilution	Use
CD19 Monoclonal Antibody (eBio1D3 (1D3)), PE	Thermo-Fisher, Waltham, Massachusetts, USA #12-0193-82	1:200	Lineage
CD45R (B220) Monoclonal Antibody (RA3-6B2), APC-eFluor™ 780	Thermo-Fisher, Waltham, Massachusetts, USA #47-0452-82	1:200	Lineage
GL7 Monoclonal Antibody (GL-7 (GL7)), Alexa Fluor™ 488	Thermo-Fisher, Waltham, Massachusetts, USA #53-5902-82	1:100	GC cell
Brilliant Violet 605™ anti-mouse CD138 (Syndecan-1)	BioLegend, San Diego, California, USA #562610	1:100	Plasma Cell

IgM Monoclonal Antibody (II/41), PE-Cyanine7	Thermo-Fisher, Waltham, Massachusetts, USA #25-5790-82	1:1600	Memory/MZ
Alexa Fluor® 647 Hamster Anti-Mouse CD95	BD Biosciences, Franklin Lakes, New Jersey, USA #563647	1:100	Memory
BV510 Rat Anti-Mouse CD23 Clone B3B4 (RUO)	BD Biosciences, Franklin Lakes, New Jersey, USA #563200	1:100	MZ B-cell
Ultra-LEAF™ Purified anti-mouse CD16/32 Antibody	BioLegend, San Diego, California, USA #101330	1:1000	Fc Block

Table S2: Antibodies used as markers for B-cell flow cytometry

Name	Manufacturer	Dilution	Use
APC/Cyanine7 anti-mouse CD45 Antibody	BioLegend, San Diego, California, USA #103116	1:100	Lineage
CD11b Monoclonal Antibody (M1/70), PE	Thermo-Fisher, Waltham, Massachusetts, USA #12-0112-82	1:400	Lineage
Alexa Fluor® 647 anti-mouse Ly-6C Antibody	BioLegend, San Diego, California, USA #128010	1:800	Classical/non-classical

Ly-6G/Ly-6C Monoclonal Antibody (RB6-8C5), FITC	Thermo-Fisher, Waltham, Massachusetts, USA #11-5931-82	1:800	Neutrophils
CD11c Monoclonal Antibody (N418), PE-Cyanine7	Thermo-Fisher, Waltham, Massachusetts, USA #25-0114-82	1:100	Dendritic Cell
Brilliant Violet 510™ anti-mouse I-A/I-E Antibody	BioLegend, San Diego, California, USA #107635	1:100	Dendritic Cell
Pacific Blue™ anti-mouse F4/80 Antibody	BioLegend, San Diego, California, USA #123123	1:100	Macrophage

Table S3: Antibodies used as markers for myeloid subsets

Name	Manufacturer	Dilution	Use
APC/Cyanine7 anti-mouse CD3 Antibody	BioLegend, San Diego, California, USA #100222	1:200	Lineage
Brilliant Violet 650™ anti-mouse CD4 Antibody	BioLegend, San Diego, California, USA #100469	1:800	Lineage
Brilliant Violet 605™ anti-mouse CD8a Antibody	BioLegend, San Diego, California, USA #100743	1:1000	Lineage

APC anti-mouse CD183 (CXCR3) Antibody	BioLegend, San Diego, California, USA #126512	1:100	Th1
Brilliant Violet 421™ anti-mouse CX3CR1 Antibody	BioLegend, San Diego, California, USA #149023	1:800	CD4/CD8 Homing Th1
FITC anti-mouse/human CD44 Antibody	BioLegend, San Diego, California, USA #103022	1:800	Memory
PE/Cyanine7 anti-mouse CD62L Antibody	BioLegend, San Diego, California, USA #104418	1:1000	Naïve/Effector
PE anti-mouse CD25 Antibody	BioLegend, San Diego, California, USA #102008	1:100	TRegs

Table S4: Antibodies used as markers for naïve and Th1 subsets

Name	Manufacturer	Dilution	Use
FITC anti-mouse CD3ε Antibody	BioLegend, San Diego, California, USA #100306	1:200	Lineage
APC anti-mouse CD4 Antibody	BioLegend, San Diego, California, USA #100516	1:1000	Lineage

APC/Cyanine7 anti-mouse CD8a Antibody	BioLegend, San Diego, California, USA #100714	1:400	Lineage
Brilliant Violet 421™ anti-mouse CD194 (CCR4) Antibody	BioLegend, San Diego, California, USA #131217	1:100	Th2
Brilliant Violet 605™ anti-mouse CD196 (CCR6) Antibody	BioLegend, San Diego, California, USA #129819	1:100	Th17
PE/Cyanine7 anti-mouse CD185 (CXCR5) Antibody	BioLegend, San Diego, California, USA #145516	1:100	T Fh
PE anti-mouse CD279 (PD-1) Antibody	BioLegend, San Diego, California, USA #109104	1:200	T Fh

Table S5: Antibodies used asmarkers for Th2 and Th17 subsets

Name	Manufacturer	Dilution	Use
FITC anti-mouse CD3ε Antibody	BioLegend, San Diego, California, USA #100305	1:200	Lineage
APC anti-mouse/human CD44 Antibody	BioLegend, San Diego, California, USA #103011	1:400	Memory
PE/Cyanine7 anti-mouse CD62L Antibody	BioLegend, San Diego, California, USA	1:400	Naïve/Effector

	#104418		
FOXP3 Monoclonal Antibody (FJK-16s), PE, eBioscience™	Thermo-Fisher, Waltham, Massachusetts, USA #12-5773-82	1:200	TRegs- intracellular
Brilliant Violet 711™ anti-mouse CD25 Antibody	BioLegend, San Diego, California, USA #102049	1:200	TRegs

Table S6: Antibodies used as markers for memory and regulatory T-cells

Histology materials

Name	Manufacturer	Dilution	Use
Purified Rat Anti-Mouse CD107b clone M3/84	BD Biosciences, Franklin Lakes, New Jersey, USA #553322	1:100	Primary antibody
Anti-Actin, α-Smooth Muscle - FITC antibody, Mouse monoclonal	Sigma-Aldrich, St. Louis, Missouri, USA #F3777	1:3000	Primary antibody
HSP70/HSPA1A Antibody	Novus Biologicals, Abingdon, UK #NB110-61582	1:20,000	Primary antibody
HSP27 Antibody - BSA Free	Novus Biologicals, Abingdon, UK #NBP1-75477	1:1000	Primary antibody
Rabbit Anti-Rat IgG Antibody, mouse adsorbed (H+L), Biotinylated	Vector Labs, Burlingame, California, USA #BA-4001	1:300	Secondary antibody for Mac-3

Biotin-SP (long spacer) IgG Fraction Monoclonal Mouse Anti-Fluorescein (FITC)	Jackson ImmunoResearch, West Grove, Pennsylvania, USA #200-062-037	1:600	Secondary antibody for αSMA
Goat Anti-Rabbit Immunoglobulins/Biotin (affinity isolated)	Dako, Santa-Clara, California, USA #E0432	1:300	Secondary antibody for Hsp27
Goat Anti-Mouse Immunoglobulins/Biotin (affinity isolated)	Dako, Santa-Clara, California, USA #E0433	1:400	Secondary antibody for Hsp70
Vectastain ABC-AP Kit, Alkaline Phosphatase (Standard)	Vector Labs, Burlingame, California, USA #AK-5000	1:50	Secondary antibody detection
Vectastain ABC-HRP kit, Peroxidase (Standard)	Vector Labs, Burlingame, California, USA #PK-4000	1:50	Secondary antibody detection
ImmPACT Vector Red Substrate	Vector Labs, Burlingame, California, USA #SK-5105	According to manufacturer	Chromogen
Vector Blue Substrate Kit, Alkaline Phosphatase (AP)	Vector Labs, Burlingame, California, USA #SK-5300	According to manufacturer	Chromogen
ImmPACT DAB Substrate, Peroxidase (HRP)	Vector Labs, Burlingame, California, USA #SK-4105	According to manufacturer	Chromogen

Antigen unmasking solution, citric acid based	Vector Labs, Burlingame, California, USA #H-3300	1:106	Antigen retrieval

Table S7: Materials used inhistology stains

Pathological assessment reveals no digestive tract abnormalities in any group but moderate to severe liver inflammation following PD fluid exposure

**Group**	**Liver**	**Stomach, small intestine, colon**	**Stomach, small intestine, colon**
Control	No inflammation	No villus abnormalities	No inflammation
CKD	No inflammation	No villus abnormalities	No inflammation
PD	Moderate to severe peritonitis	No villus abnormalities	Severe peritonitis and inflammatory cell infiltration

Table S8: Pathological summary of liver and digestive tract. A singled blinded operator assessed liver and digestive tract inflammation, and searched for abnormalities in the villi of the stomach, small intestine and colon.

Interventions are well tolerated in experimental mice

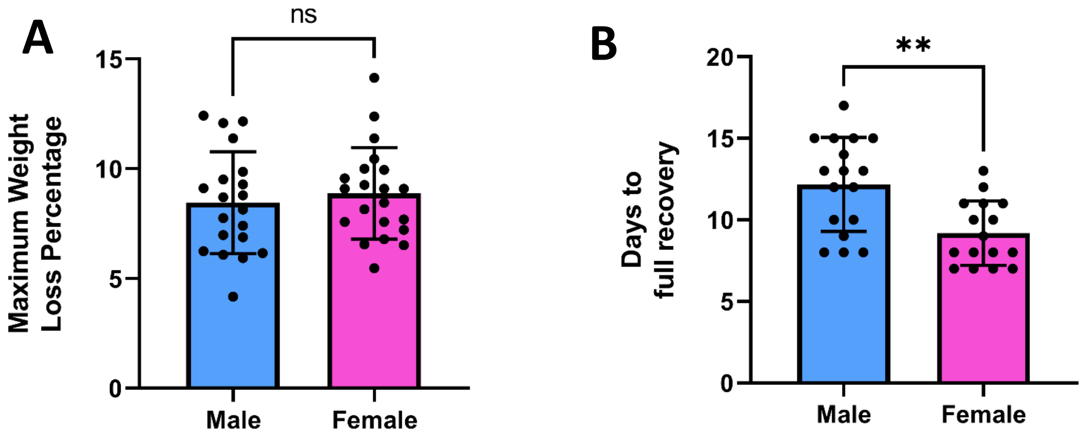

Figure S1: Male mice are slower to return to their pre-operative body weight following 5/6 nephrectomy surgery. Male and female mice were subjected to 5/6 nephrectomy, and their mean maximum weight loss from pre-surgical weight measurement (A) and days taken to return to that body weight following surgery (B) were recorded. Mean value with standard deviation shown, n=20. ** p ≤0.01.

T-cell gating strategy

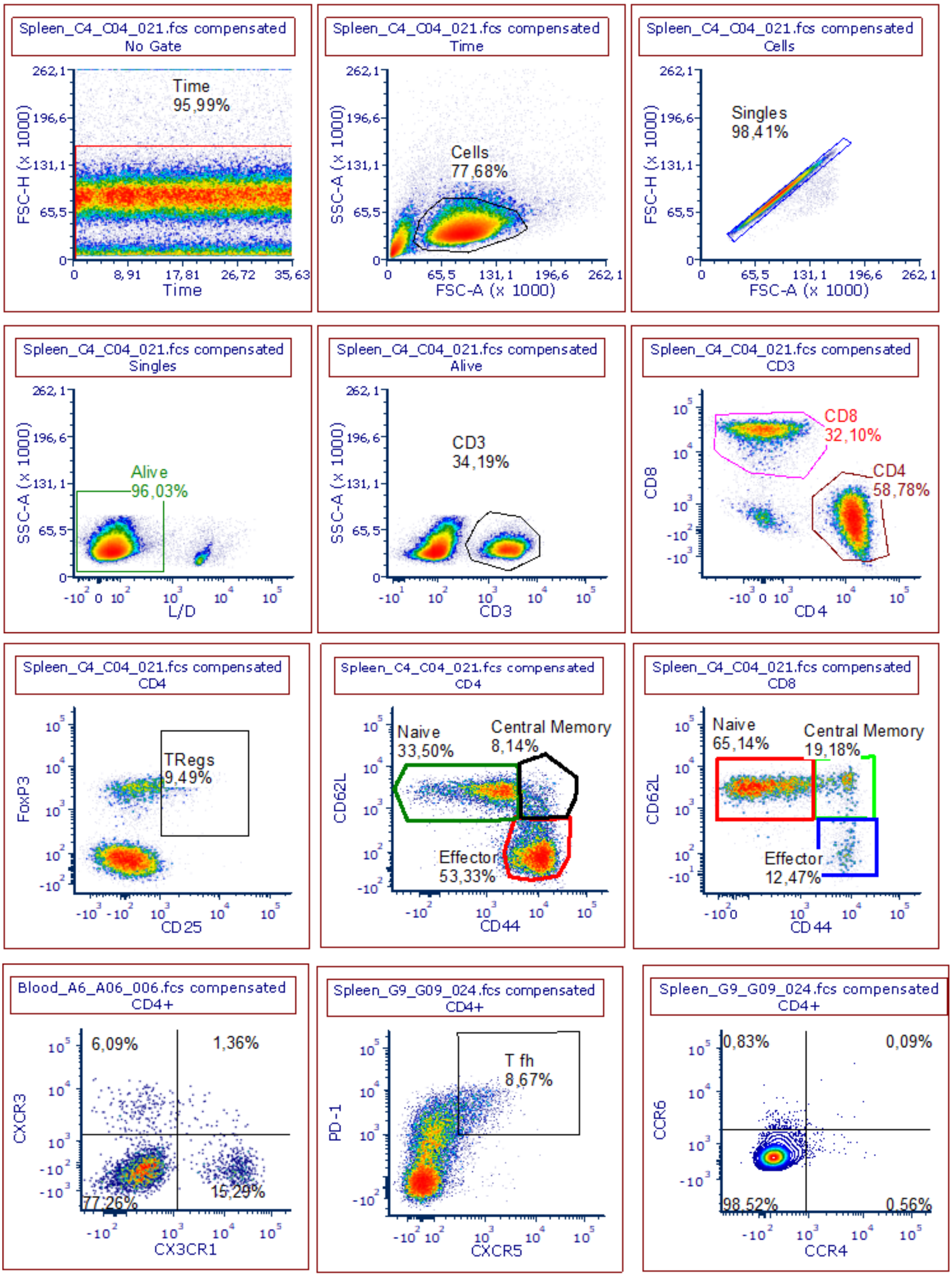

Figure S2: Gating strategy for analysis of T-cell flow cytometry data is shown

Myeloid gating strategy

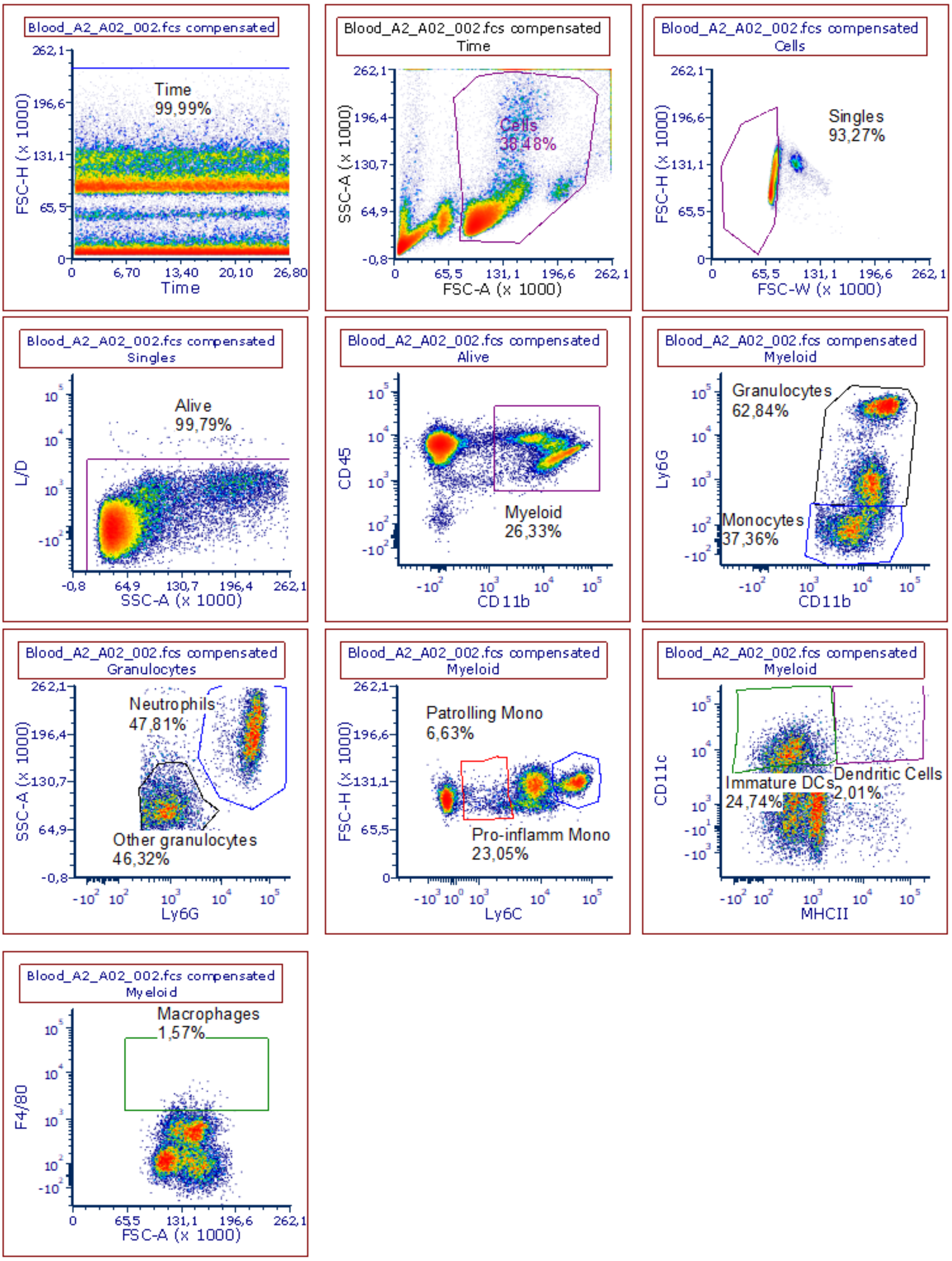

Figure S3: Gating strategy for analysis of myeloid flow cytometry data is shown

B-cell gating strategy

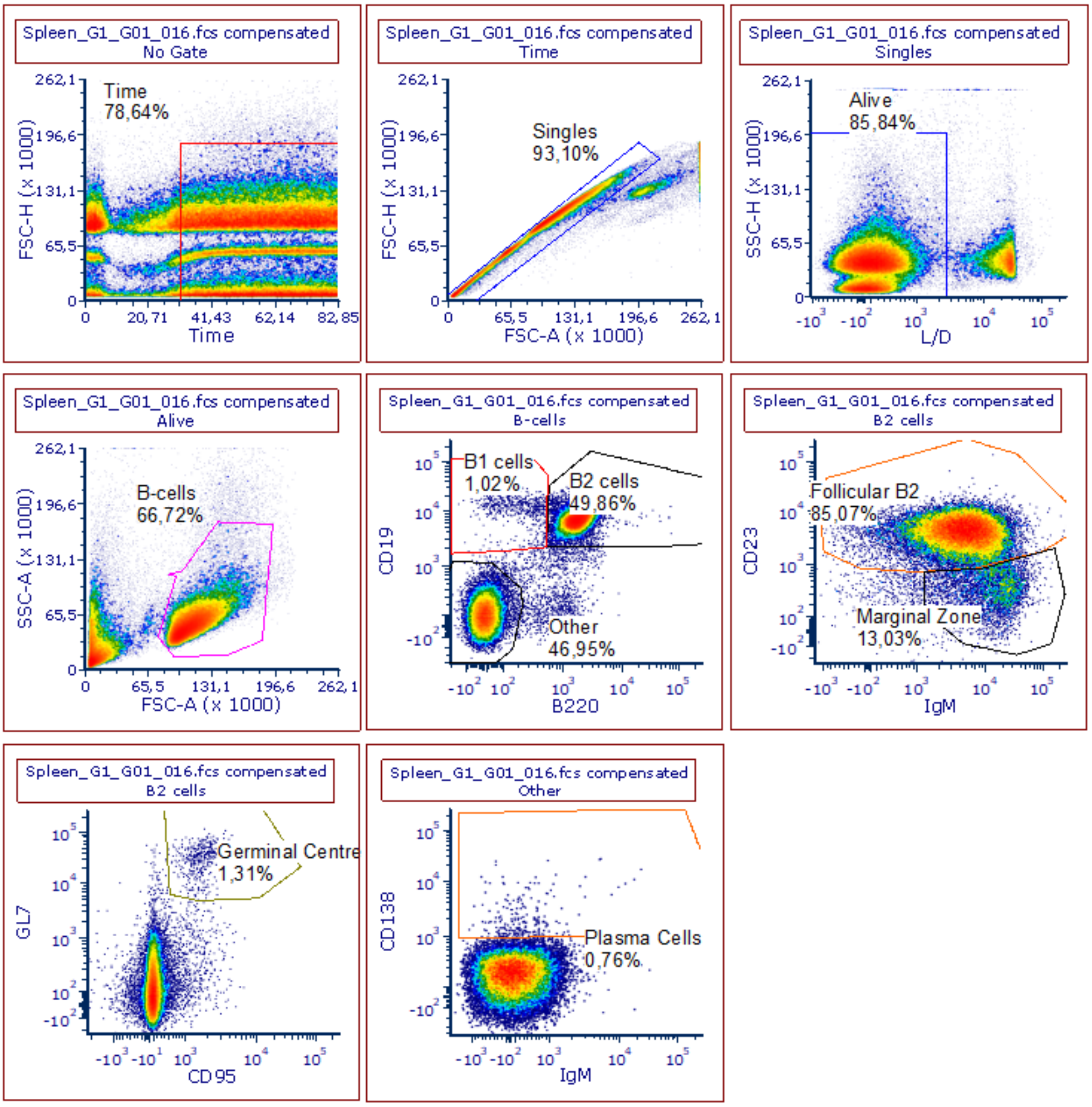

Figure S4: Gating strategy for analysis of B-cell flow cytometry data is shown

Plaque histology readouts are unchanged following induced chronic kidney disease and peritoneal dialysis fluid exposure

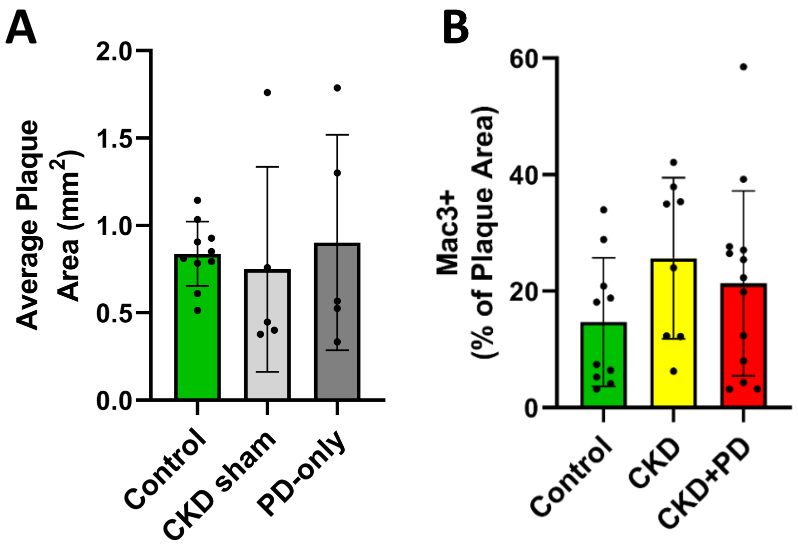

Figure S5: Plaque area in sham mice and absolute macrophage content is unchanged in the aortic arch of mice exposed to induced CKD or induced CKD combined with peritoneal dialysis fluid exposure. (A) mean plaque area in the aortic arch with SD, n=13, n=5, n=5. **(B)** Percentage of plaque area positive for Mac3 is shown with SD. n=9.

Chronic kidney disease and peritoneal dialysis does not change the T-cell composition of several subsets in the blood and spleen

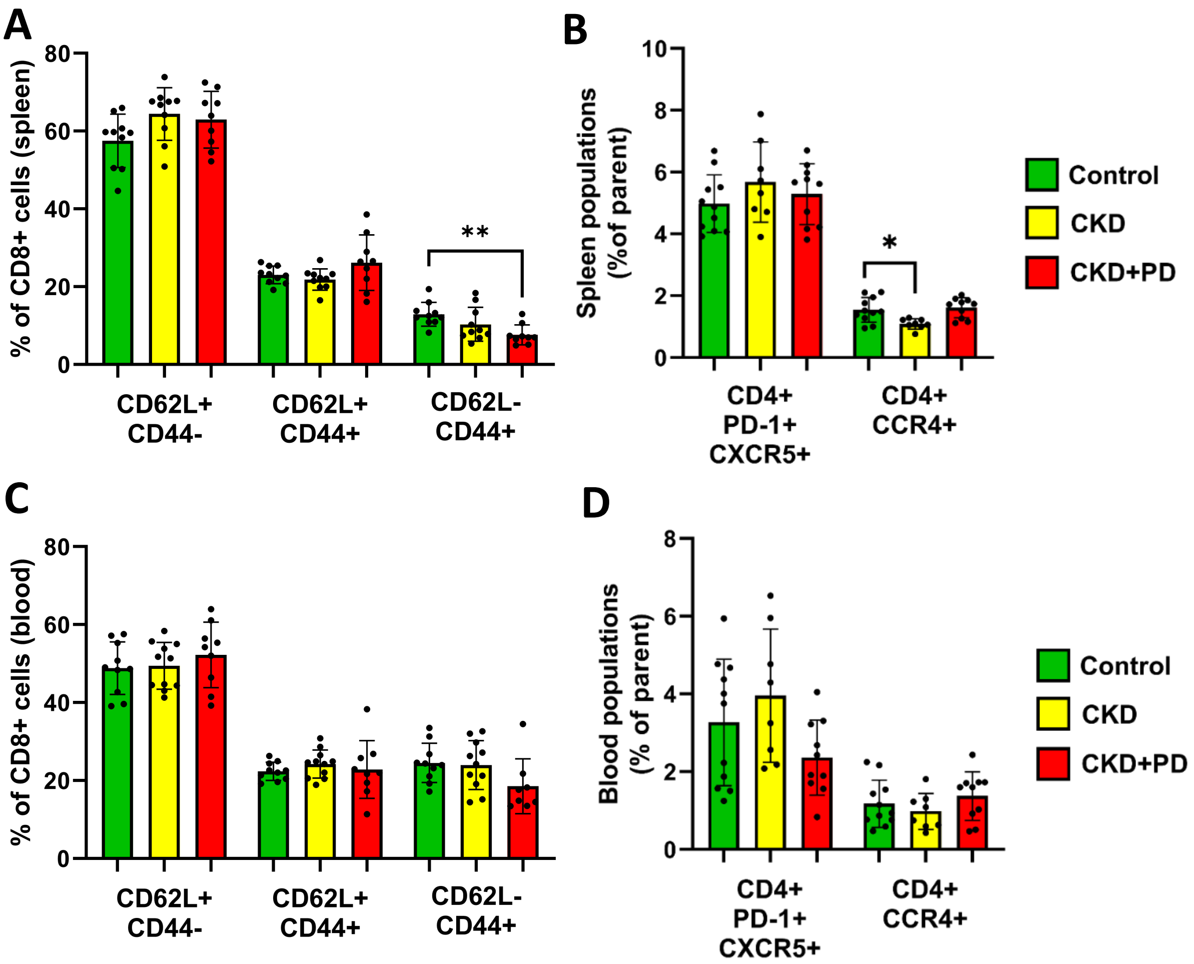

Figure S6: Response of T-cell immune subsets following induced chronic kidney disease and peritoneal dialysis fluid exposure. Percentage of **(A)** spleen populations containing stated $CD8^+$ immune subsets and **(B)** spleen populations containing stated $CD4^+$ immune subsets. Percentage of **(A)** blood populations containing stated $CD8^+$ immune subsets and **(B)** blood populations containing stated $CD4^+$ immune subsets. ** $p \leq 0.01$. n=9.

Chronic kidney disease and peritoneal dialysis does not change the T-cell composition of the mesenteric lymph node

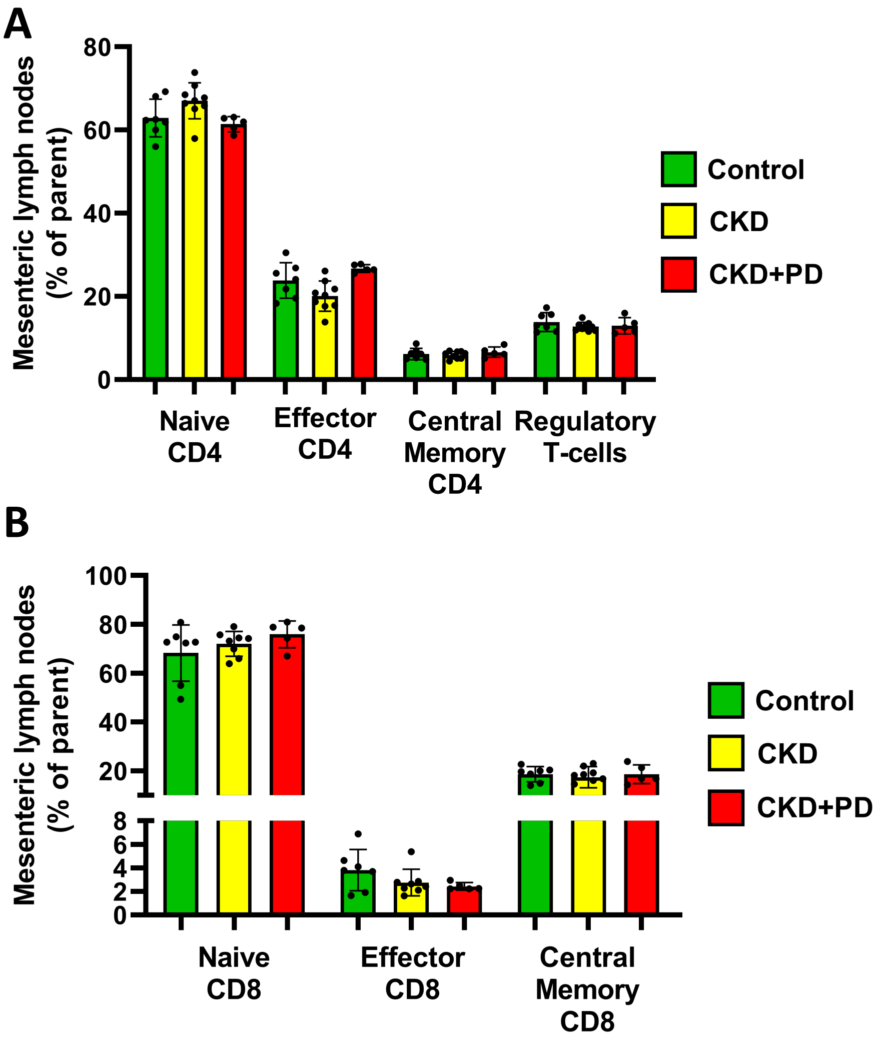

Figure S7: Response of T-cell immune subsets following induced chronic kidney disease and peritoneal dialysis fluid exposure in the mesenteric lymph node. Percentage of parent population containing stated immune subsets in the mesenteric lymph nodes are shown **(A and B)**. n=9.

Chronic kidney disease and peritoneal dialysis does not change several myeloid cells in the spleen and blood

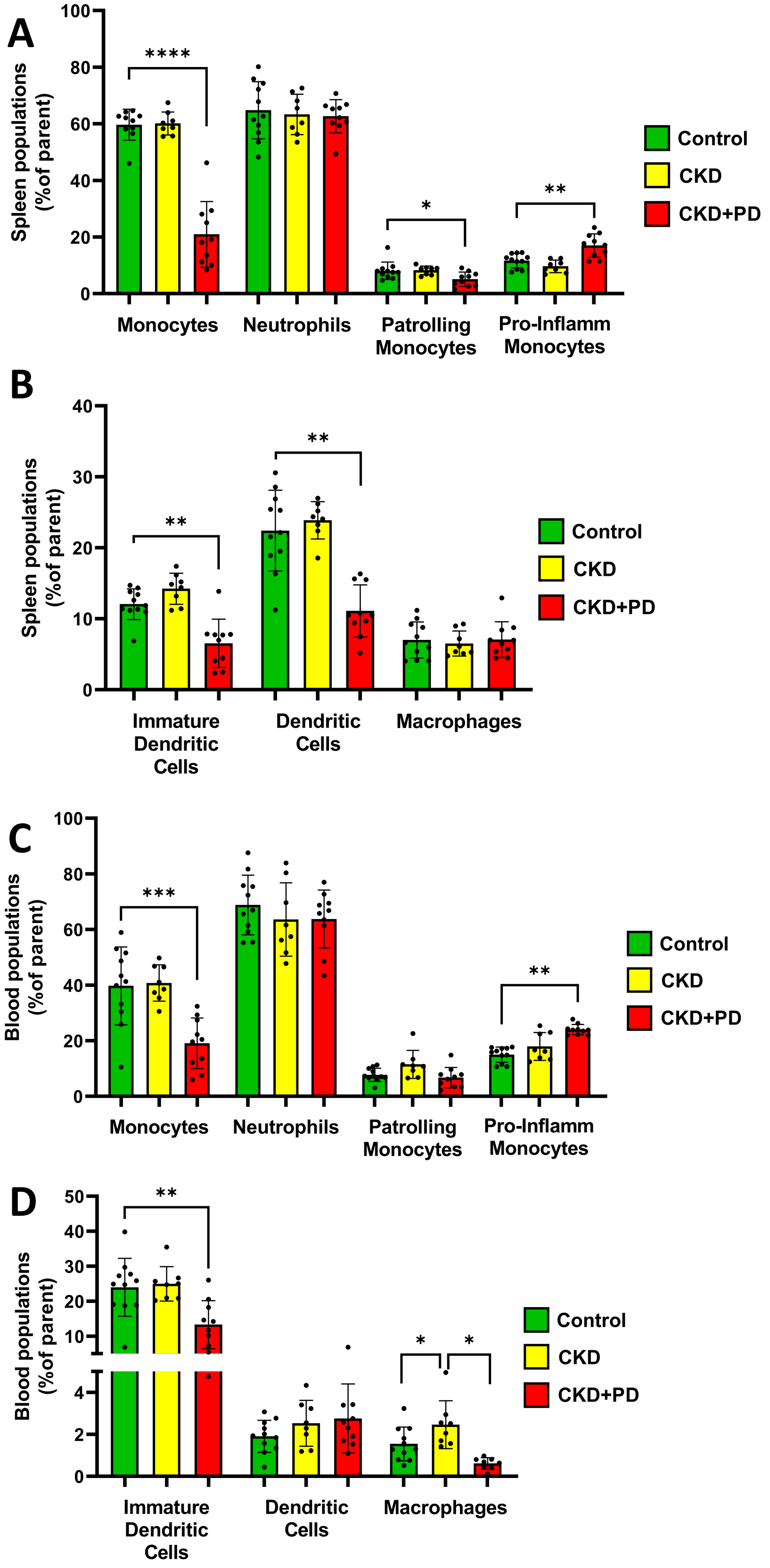

Figure S8: Myeloid cells in the spleen and blood are shown followinginduced chronic kidney disease and peritoneal dialysis. Percentage of parent population containing stated immune subsets in the spleen **(A and B)** and blood **(C and D)** are shown. * $p \leq 0.05$. ** $p \leq 0.01$. *** $p \leq 0.001$. **** $p \leq 0.0001$. n=9.

Chronic kidney disease and peritoneal dialysis exposure does not change B-cell populations in spleen or blood

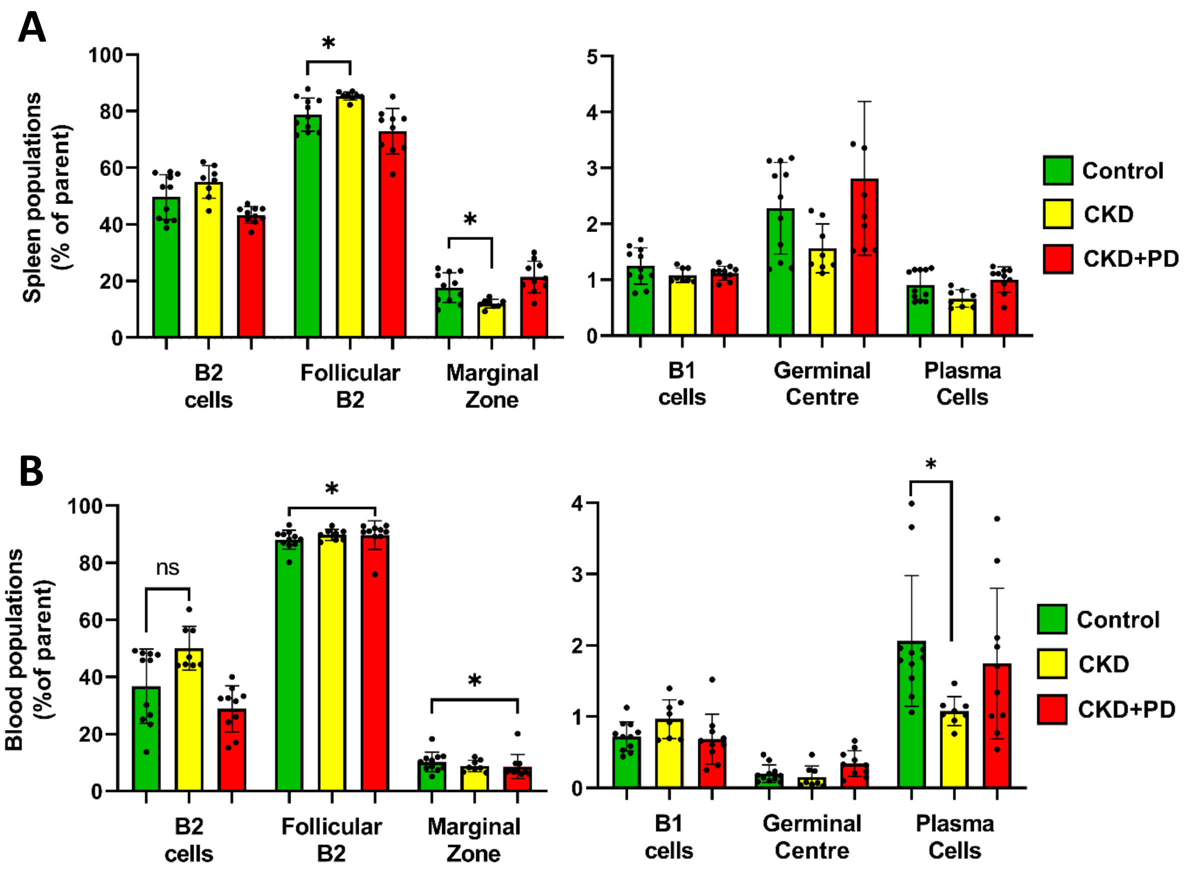

Figure S9: B-cell populations in the spleen and blood are shown following induced chronic kidney disease and peritoneal dialysis. Percentage of parent population containing stated immune subsets in the spleen **(A)** and blood **(B)** are shown. * $p \leq 0.05$. n=9.

Liver collagen and cholesterol content is unchanged

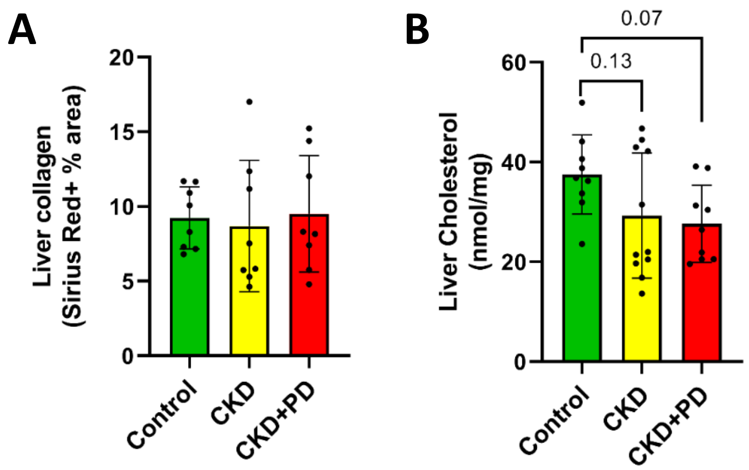

Figure S10: Liver collagen and liver cholesterol content is unchanged. **(A)** Liver collagen content was assessed via picrosirius red staining **(B)** liver cholesterol concentrations were assessed. n=9.
